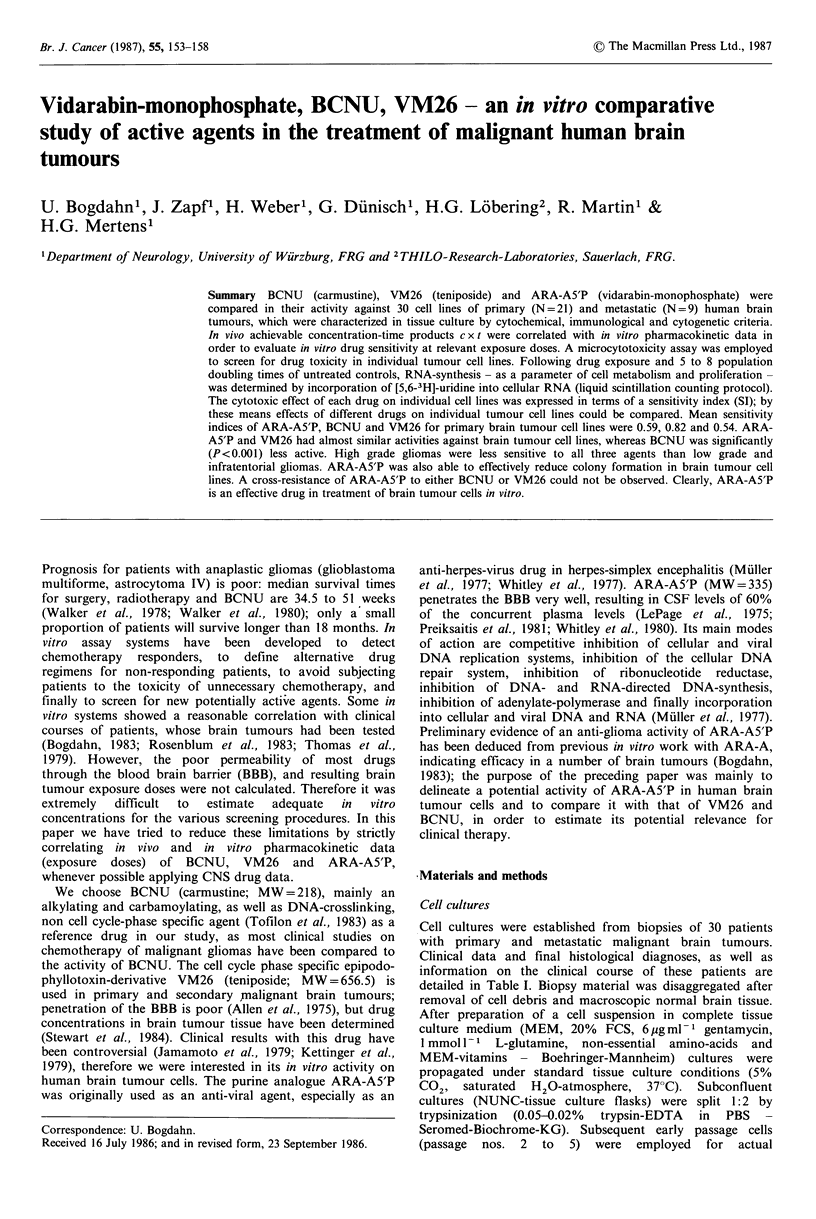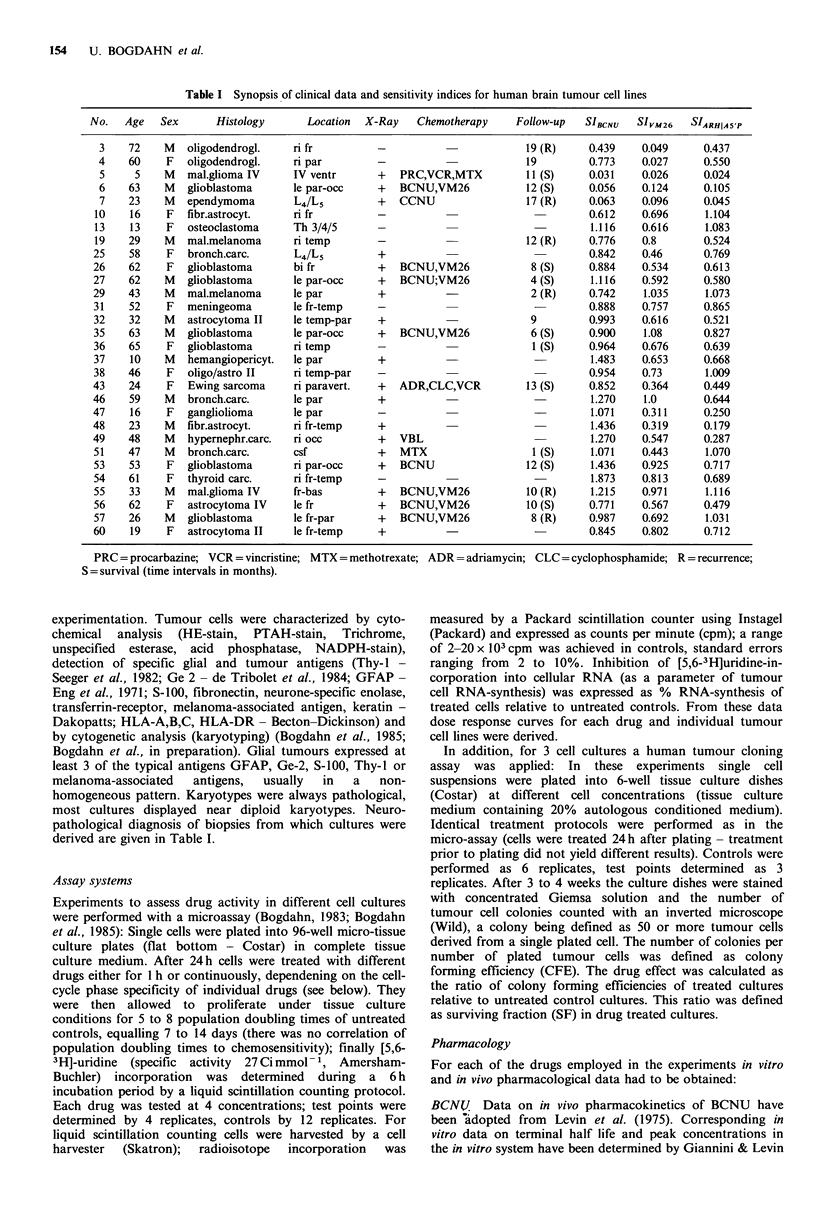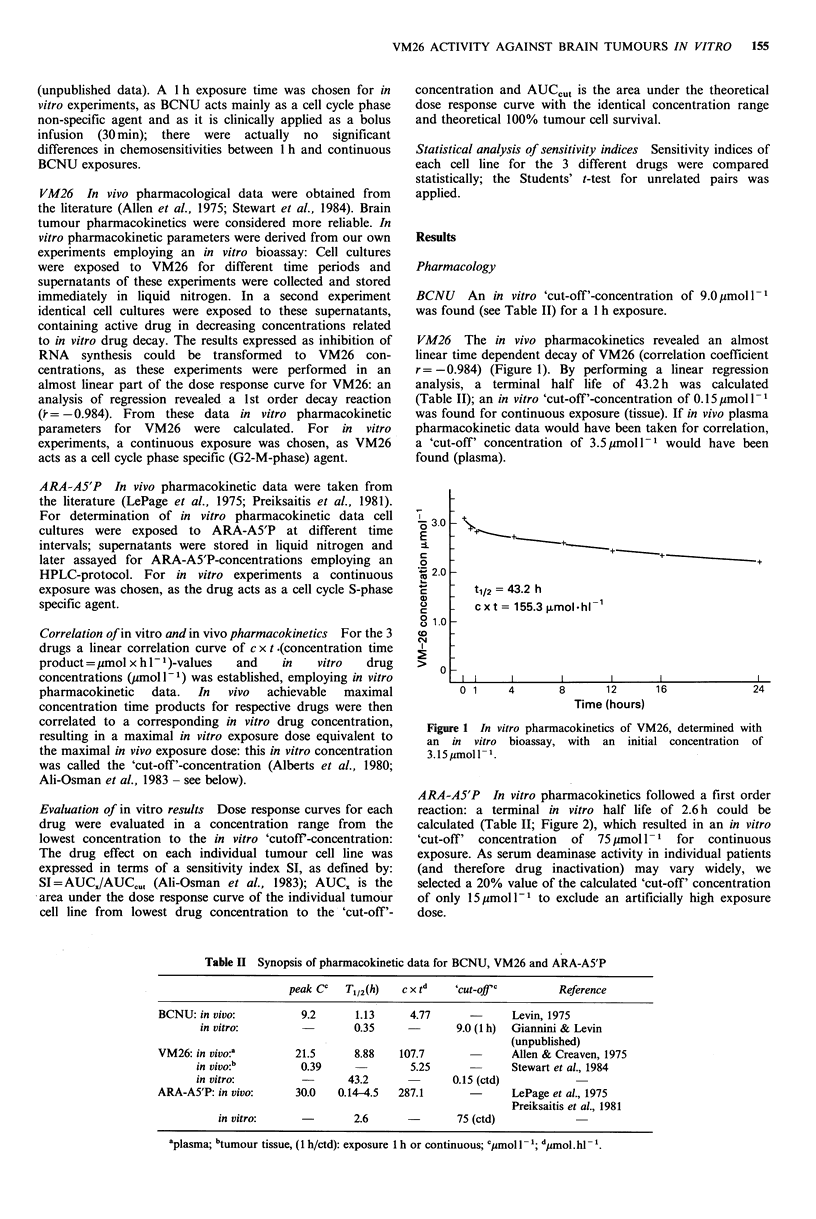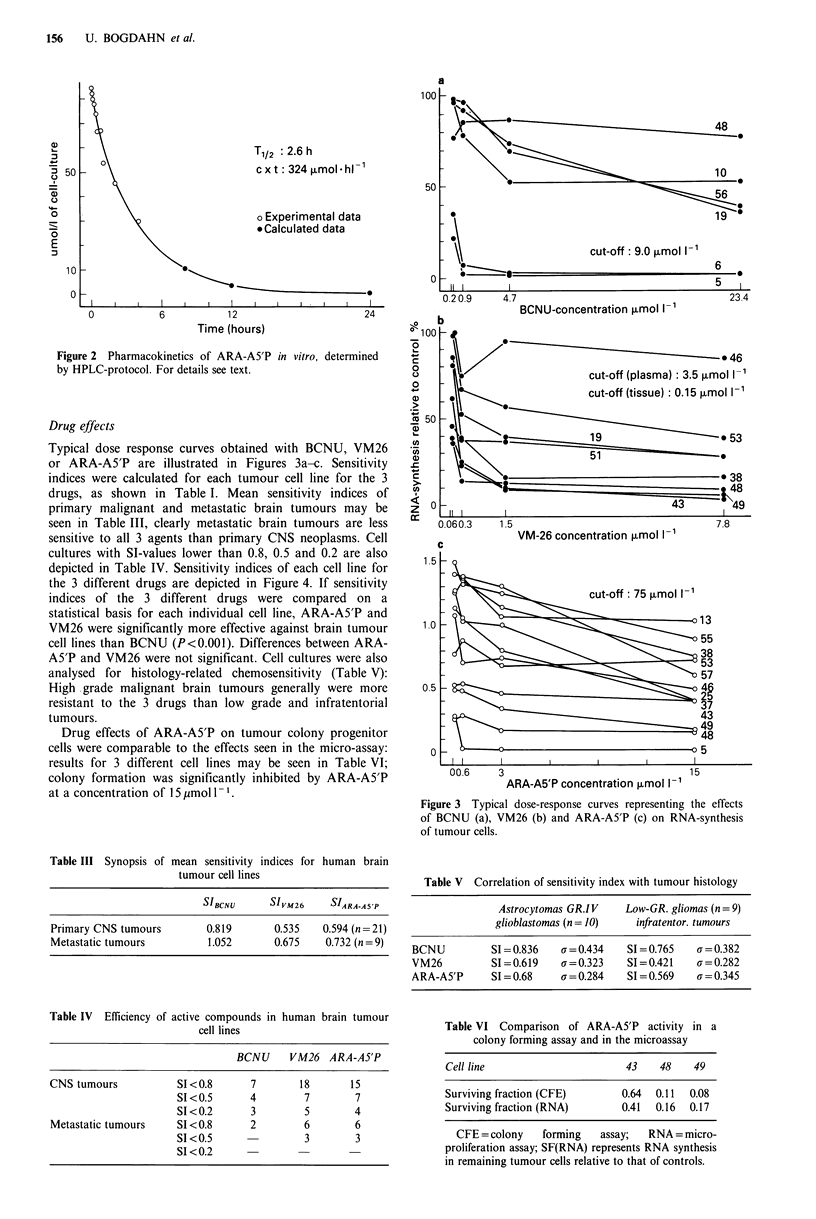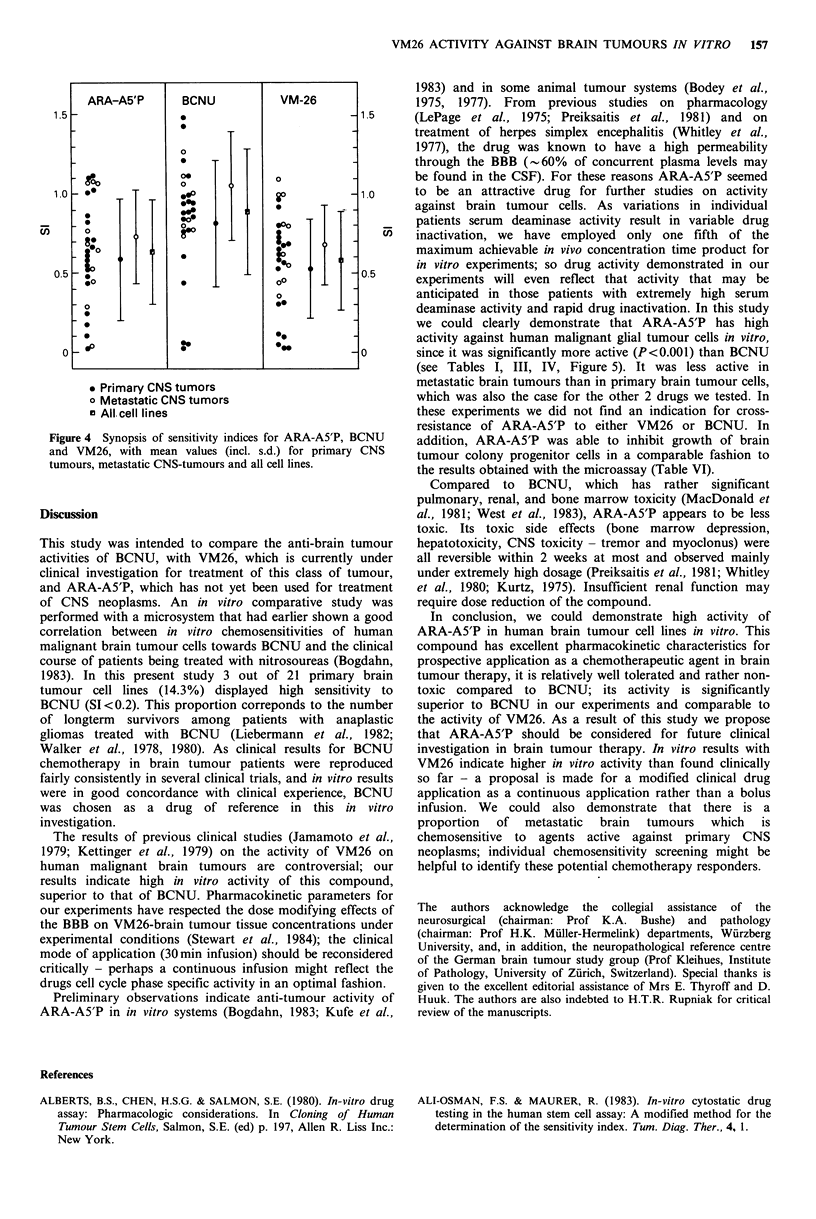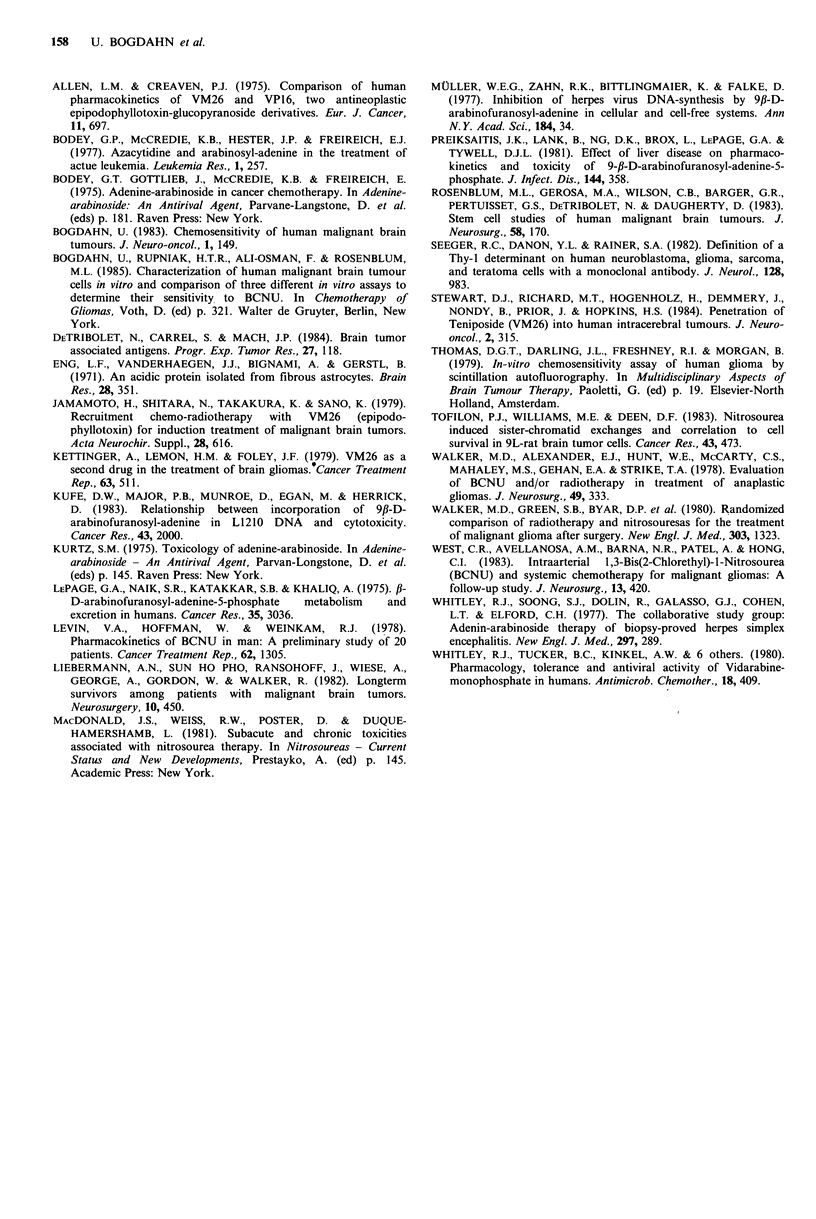# Vidarabin-monophosphate, BCNU, VM26--an in vitro comparative study of active agents in the treatment of malignant human brain tumours.

**DOI:** 10.1038/bjc.1987.31

**Published:** 1987-02

**Authors:** U. Bogdahn, J. Zapf, H. Weber, G. Dünisch, H. G. Löbering, R. Martin, H. G. Mertens

## Abstract

BCNU (carmustine), VM26 (teniposide) and ARA-A5'P (vidarabin-monophosphate) were compared in their activity against 30 cell lines of primary (N = 21) and metastatic (N = 9) human brain tumours, which were characterized in tissue culture by cytochemical, immunological and cytogenetic criteria. In vivo achievable concentration-time products c X t were correlated with in vitro pharmacokinetic data in order to evaluate in vitro drug sensitivity at relevant exposure doses. A microcytotoxicity assay was employed to screen for drug toxicity in individual tumour cell lines. Following drug exposure and 5 to 8 population doubling times of untreated controls, RNA-synthesis - as a parameter of cell metabolism and proliferation - was determined by incorporation of [5,6-3H]-uridine into cellular RNA (liquid scintillation counting protocol). The cytotoxic effect of each drug on individual cell lines was expressed in terms of a sensitivity index (SI); by these means effects of different drugs on individual tumour cell lines could be compared. Mean sensitivity indices of ARA-A5'P, BCNU and VM26 for primary brain tumour cell lines were 0.59, 0.82 and 0.54. ARA-A5'P and VM26 had almost similar activities against brain tumour cell lines, whereas BCNU was significantly (P less than 0.001) less active. High grade gliomas were less sensitive to all three agents than low grade and infratentorial gliomas. ARA-A5'P was also able to effectively reduce colony formation in brain tumour cell lines. A cross-resistance of ARA-A5'P to either BCNU or VM26 could not be observed. Clearly, ARA-A5'P is an effective drug in treatment of brain tumour cells in vitro.


*Br. J. Cancer* (1987), **55**, 153–158 © The Macmillan Press Ltd., 1987

### Vidarabin-monophosphate, BCNU, VM26 – an *in vitro* comparative study of active agents in the treatment of malignant human brain tumours

U. Bogdahn[1], J. Zapf[1], H. Weber[1], G. Dünisch[1], H.G. Löbering[2], R. Martin[1] & H.G. Mertens[1]

[1]*Department of Neurology, University of Würzburg, FRG and* [2]*THILO-Research-Laboratories, Sauerlach, FRG.*

**Summary** BCNU (carmustine), VM26 (teniposide) and ARA-A5′P (vidarabin-monophosphate) were compared in their activity against 30 cell lines of primary (N=21) and metastatic (N=9) human brain tumours, which were characterized in tissue culture by cytochemical, immunological and cytogenetic criteria. *In vivo* achievable concentration-time products $c \times t$ were correlated with *in vitro* pharmacokinetic data in order to evaluate *in vitro* drug sensitivity at relevant exposure doses. A microcytotoxicity assay was employed to screen for drug toxicity in individual tumour cell lines. Following drug exposure and 5 to 8 population doubling times of untreated controls, RNA-synthesis – as a parameter of cell metabolism and proliferation – was determined by incorporation of [5,6-$^3$H]-uridine into cellular RNA (liquid scintillation counting protocol). The cytotoxic effect of each drug on individual cell lines was expressed in terms of a sensitivity index (SI); by these means effects of different drugs on individual tumour cell lines could be compared. Mean sensitivity indices of ARA-A5′P, BCNU and VM26 for primary brain tumour cell lines were 0.59, 0.82 and 0.54. ARA-A5′P and VM26 had almost similar activities against brain tumour cell lines, whereas BCNU was significantly ($P<0.001$) less active. High grade gliomas were less sensitive to all three agents than low grade and infratentorial gliomas. ARA-A5′P was also able to effectively reduce colony formation in brain tumour cell lines. A cross-resistance of ARA-A5′P to either BCNU or VM26 could not be observed. Clearly, ARA-A5′P is an effective drug in treatment of brain tumour cells *in vitro*.

Prognosis for patients with anaplastic gliomas (glioblastoma multiforme, astrocytoma IV) is poor: median survival times for surgery, radiotherapy and BCNU are 34.5 to 51 weeks (Walker *et al.*, 1978; Walker *et al.*, 1980); only a small proportion of patients will survive longer than 18 months. *In vitro* assay systems have been developed to detect chemotherapy responders, to define alternative drug regimens for non-responding patients, to avoid subjecting patients to the toxicity of unnecessary chemotherapy, and finally to screen for new potentially active agents. Some *in vitro* systems showed a reasonable correlation with clinical courses of patients, whose brain tumours had been tested (Bogdahn, 1983; Rosenblum *et al.*, 1983; Thomas *et al.*, 1979). However, the poor permeability of most drugs through the blood brain barrier (BBB), and resulting brain tumour exposure doses were not calculated. Therefore it was extremely difficult to estimate adequate *in vitro* concentrations for the various screening procedures. In this paper we have tried to reduce these limitations by strictly correlating *in vivo* and *in vitro* pharmacokinetic data (exposure doses) of BCNU, VM26 and ARA-A5′P, whenever possible applying CNS drug data.

We choose BCNU (carmustine; MW=218), mainly an alkylating and carbamoylating, as well as DNA-crosslinking, non cell cycle-phase specific agent (Tofilon *et al.*, 1983) as a reference drug in our study, as most clinical studies on chemotherapy of malignant gliomas have been compared to the activity of BCNU. The cell cycle phase specific epipodophyllotoxin-derivative VM26 (teniposide; MW=656.5) is used in primary and secondary malignant brain tumours; penetration of the BBB is poor (Allen *et al.*, 1975), but drug concentrations in brain tumour tissue have been determined (Stewart *et al.*, 1984). Clinical results with this drug have been controversial (Jamamoto *et al.*, 1979; Kettinger *et al.*, 1979), therefore we were interested in its *in vitro* activity on human brain tumour cells. The purine analogue ARA-A5′P was originally used as an anti-viral agent, especially as an anti-herpes-virus drug in herpes-simplex encephalitis (Müller *et al.*, 1977; Whitley *et al.*, 1977). ARA-A5′P (MW=335) penetrates the BBB very well, resulting in CSF levels of 60% of the concurrent plasma levels (LePage *et al.*, 1975; Preiksaitis *et al.*, 1981; Whitley *et al.*, 1980). Its main modes of action are competitive inhibition of cellular and viral DNA replication systems, inhibition of the cellular DNA repair system, inhibition of ribonucleotide reductase, inhibition of DNA- and RNA-directed DNA-synthesis, inhibition of adenylate-polymerase and finally incorporation into cellular and viral DNA and RNA (Müller *et al.*, 1977). Preliminary evidence of an anti-glioma activity of ARA-A5′P has been deduced from previous *in vitro* work with ARA-A, indicating efficacy in a number of brain tumours (Bogdahn, 1983); the purpose of the preceding paper was mainly to delineate a potential activity of ARA-A5′P in human brain tumour cells and to compare it with that of VM26 and BCNU, in order to estimate its potential relevance for clinical therapy.

### Materials and methods

### *Cell cultures*

Cell cultures were established from biopsies of 30 patients with primary and metastatic malignant brain tumours. Clinical data and final histological diagnoses, as well as information on the clinical course of these patients are detailed in Table I. Biopsy material was disaggregated after removal of cell debris and macroscopic normal brain tissue. After preparation of a cell suspension in complete tissue culture medium (MEM, 20% FCS, 6 $\mu$g ml$^{-1}$ gentamycin, 1 mmol l$^{-1}$ L-glutamine, non-essential amino-acids and MEM-vitamins – Boehringer-Mannheim) cultures were propagated under standard tissue culture conditions (5% $CO_2$, saturated $H_2O$-atmosphere, 37°C). Subconfluent cultures (NUNC-tissue culture flasks) were split 1:2 by trypsinization (0.05–0.02% trypsin-EDTA in PBS – Seromed-Biochrome-KG). Subsequent early passage cells (passage nos. 2 to 5) were employed for actual

Correspondence: U. Bogdahn.
Received 16 July 1986; and in revised form, 23 September 1986.

Table I Synopsis of clinical data and sensitivity indices for human brain tumour cell lines

| No. | Age | Sex | Histology | Location | X-Ray | Chemotherapy | Follow-up | $SI_{BCNU}$ | $SI_{VM26}$ | $SI_{ARH\|AS'P}$ |
|---|---|---|---|---|---|---|---|---|---|---|
| 3 | 72 | M | oligodendrogl. | ri fr | – | — | 19 (R) | 0.439 | 0.049 | 0.437 |
| 4 | 60 | F | oligodendrogl. | ri par | – | — | 19 | 0.773 | 0.027 | 0.550 |
| 5 | 5 | M | mal.glioma IV | IV ventr | + | PRC,VCR,MTX | 11 (S) | 0.031 | 0.026 | 0.024 |
| 6 | 63 | M | glioblastoma | le par-occ | + | BCNU,VM26 | 12 (S) | 0.056 | 0.124 | 0.105 |
| 7 | 23 | M | ependymoma | $L_4/L_5$ | + | CCNU | 17 (R) | 0.063 | 0.096 | 0.045 |
| 10 | 16 | F | fibr.astrocyt. | ri fr | – | — | — | 0.612 | 0.696 | 1.104 |
| 13 | 13 | F | osteoclastoma | Th 3/4/5 | – | — | — | 1.116 | 0.616 | 1.083 |
| 19 | 29 | M | mal.melanoma | ri temp | – | — | 12 (R) | 0.776 | 0.8 | 0.524 |
| 25 | 58 | F | bronch.carc. | $L_4/L_5$ | + | — | — | 0.842 | 0.46 | 0.769 |
| 26 | 62 | F | glioblastoma | bi fr | + | BCNU,VM26 | 8 (S) | 0.884 | 0.534 | 0.613 |
| 27 | 62 | M | glioblastoma | le par-occ | + | BCNU;VM26 | 4 (S) | 1.116 | 0.592 | 0.580 |
| 29 | 43 | M | mal.melanoma | le par | + | — | 2 (R) | 0.742 | 1.035 | 1.073 |
| 31 | 52 | F | meningeoma | le fr-temp | – | — | — | 0.888 | 0.757 | 0.865 |
| 32 | 32 | M | astrocytoma II | le temp-par | + | — | 9 | 0.993 | 0.616 | 0.521 |
| 35 | 63 | M | glioblastoma | le par-occ | + | BCNU,VM26 | 6 (S) | 0.900 | 1.08 | 0.827 |
| 36 | 65 | F | glioblastoma | ri temp | – | — | 1 (S) | 0.964 | 0.676 | 0.639 |
| 37 | 10 | M | hemangiopericyt. | le par | + | — | — | 1.483 | 0.653 | 0.668 |
| 38 | 46 | F | oligo/astro II | ri temp-par | – | — | — | 0.954 | 0.73 | 1.009 |
| 43 | 24 | F | Ewing sarcoma | ri paravert. | + | ADR,CLC,VCR | 13 (S) | 0.852 | 0.364 | 0.449 |
| 46 | 59 | M | bronch.carc. | le par | + | — | — | 1.270 | 1.0 | 0.644 |
| 47 | 16 | F | gangliolioma | le par | – | — | — | 1.071 | 0.311 | 0.250 |
| 48 | 23 | M | fibr.astrocyt. | ri fr-temp | + | — | — | 1.436 | 0.319 | 0.179 |
| 49 | 48 | M | hypernephr.carc. | ri occ | + | VBL | — | 1.270 | 0.547 | 0.287 |
| 51 | 47 | M | bronch.carc. | csf | + | MTX | 1 (S) | 1.071 | 0.443 | 1.070 |
| 53 | 53 | F | glioblastoma | ri par-occ | + | BCNU | 12 (S) | 1.436 | 0.925 | 0.717 |
| 54 | 61 | F | thyroid carc. | ri fr-temp | – | — | — | 1.873 | 0.813 | 0.689 |
| 55 | 33 | M | mal.glioma IV | fr-bas | + | BCNU,VM26 | 10 (R) | 1.215 | 0.971 | 1.116 |
| 56 | 62 | F | astrocytoma IV | le fr | + | BCNU,VM26 | 10 (S) | 0.771 | 0.567 | 0.479 |
| 57 | 26 | M | glioblastoma | le fr-par | + | BCNU,VM26 | 8 (R) | 0.987 | 0.692 | 1.031 |
| 60 | 19 | F | astrocytoma II | le fr-temp | + | — | — | 0.845 | 0.802 | 0.712 |

PRC = procarbazine; VCR = vincristine; MTX = methotrexate; ADR = adriamycin; CLC = cyclophosphamide; R = recurrence; S = survival (time intervals in months).

experimentation. Tumour cells were characterized by cytochemical analysis (HE-stain, PTAH-stain, Trichrome, unspecified esterase, acid phosphatase, NADPH-stain), detection of specific glial and tumour antigens (Thy-1 – Seeger *et al.*, 1982; Ge 2 – de Tribolet *et al.*, 1984; GFAP – Eng *et al.*, 1971; S-100, fibronectin, neurone-specific enolase, transferrin-receptor, melanoma-associated antigen, keratin – Dakopatts; HLA-A,B,C, HLA-DR – Becton–Dickinson) and by cytogenetic analysis (karyotyping) (Bogdahn *et al.*, 1985; Bogdahn *et al.*, in preparation). Glial tumours expressed at least 3 of the typical antigens GFAP, Ge-2, S-100, Thy-1 or melanoma-associated antigens, usually in a non-homogeneous pattern. Karyotypes were always pathological, most cultures displayed near diploid karyotypes. Neuropathological diagnosis of biopsies from which cultures were derived are given in Table I.

*Assay systems*

Experiments to assess drug activity in different cell cultures were performed with a microassay (Bogdahn, 1983; Bogdahn *et al.*, 1985): Single cells were plated into 96-well micro-tissue culture plates (flat bottom – Costar) in complete tissue culture medium. After 24 h cells were treated with different drugs either for 1 h or continuously, dependening on the cell-cycle phase specificity of individual drugs (see below). They were then allowed to proliferate under tissue culture conditions for 5 to 8 population doubling times of untreated controls, equalling 7 to 14 days (there was no correlation of population doubling times to chemosensitivity); finally [5,6-$^3$H]-uridine (specific activity 27 Ci mmol$^{-1}$, Amersham-Buchler) incorporation was determined during a 6 h incubation period by a liquid scintillation counting protocol. Each drug was tested at 4 concentrations; test points were determined by 4 replicates, controls by 12 replicates. For liquid scintillation counting cells were harvested by a cell harvester (Skatron); radioisotope incorporation was measured by a Packard scintillation counter using Instagel (Packard) and expressed as counts per minute (cpm); a range of 2–20 x 10$^3$ cpm was achieved in controls, standard errors ranging from 2 to 10%. Inhibition of [5,6-$^3$H]uridine-incorporation into cellular RNA (as a parameter of tumour cell RNA-synthesis) was expressed as % RNA-synthesis of treated cells relative to untreated controls. From these data dose response curves for each drug and individual tumour cell lines were derived.

In addition, for 3 cell cultures a human tumour cloning assay was applied: In these experiments single cell suspensions were plated into 6-well tissue culture dishes (Costar) at different cell concentrations (tissue culture medium containing 20% autologous conditioned medium). Identical treatment protocols were performed as in the micro-assay (cells were treated 24 h after plating – treatment prior to plating did not yield different results). Controls were performed as 6 replicates, test points determined as 3 replicates. After 3 to 4 weeks the culture dishes were stained with concentrated Giemsa solution and the number of tumour cell colonies counted with an inverted microscope (Wild), a colony being defined as 50 or more tumour cells derived from a single plated cell. The number of colonies per number of plated tumour cells was defined as colony forming efficiency (CFE). The drug effect was calculated as the ratio of colony forming efficiencies of treated cultures relative to untreated control cultures. This ratio was defined as surviving fraction (SF) in drug treated cultures.

*Pharmacology*

For each of the drugs employed in the experiments *in vitro* and *in vivo* pharmacological data had to be obtained:

*BCNU* Data on *in vivo* pharmacokinetics of BCNU have been adopted from Levin *et al.* (1975). Corresponding *in vitro* data on terminal half life and peak concentrations in the *in vitro* system have been determined by Giannini & Levin

(unpublished data). A 1 h exposure time was chosen for *in vitro* experiments, as BCNU acts mainly as a cell cycle phase non-specific agent and as it is clinically applied as a bolus infusion (30 min); there were actually no significant differences in chemosensitivities between 1 h and continuous BCNU exposures.

*VM26* *In vivo* pharmacological data were obtained from the literature (Allen *et al.*, 1975; Stewart *et al.*, 1984). Brain tumour pharmacokinetics were considered more reliable. *In vitro* pharmacokinetic parameters were derived from our own experiments employing an *in vitro* bioassay: Cell cultures were exposed to VM26 for different time periods and supernatants of these experiments were collected and stored immediately in liquid nitrogen. In a second experiment identical cell cultures were exposed to these supernatants, containing active drug in decreasing concentrations related to *in vitro* drug decay. The results expressed as inhibition of RNA synthesis could be transformed to VM26 concentrations, as these experiments were performed in an almost linear part of the dose response curve for VM26: an analysis of regression revealed a 1st order decay reaction ($r = -0.984$). From these data *in vitro* pharmacokinetic parameters for VM26 were calculated. For *in vitro* experiments, a continuous exposure was chosen, as VM26 acts as a cell cycle phase specific (G2-M-phase) agent.

*ARA-A5′P* *In vivo* pharmacokinetic data were taken from the literature (LePage *et al.*, 1975; Preiksaitis *et al.*, 1981). For determination of *in vitro* pharmacokinetic data cell cultures were exposed to ARA-A5′P at different time intervals; supernatants were stored in liquid nitrogen and later assayed for ARA-A5′P-concentrations employing an HPLC-protocol. For *in vitro* experiments a continuous exposure was chosen, as the drug acts as a cell cycle S-phase specific agent.

*Correlation of* in vitro *and* in vivo *pharmacokinetics* For the 3 drugs a linear correlation curve of $c \times t$ -(concentration time product = $\mu$mol x h $l^{-1}$)-values and *in vitro* drug concentrations ($\mu$mol $l^{-1}$) was established, employing *in vitro* pharmacokinetic data. *In vivo* achievable maximal concentration time products for respective drugs were then correlated to a corresponding *in vitro* drug concentration, resulting in a maximal *in vitro* exposure dose equivalent to the maximal *in vivo* exposure dose: this *in vitro* concentration was called the 'cut-off'-concentration (Alberts *et al.*, 1980; Ali-Osman *et al.*, 1983 – see below).

*Evaluation of* in vitro *results* Dose response curves for each drug were evaluated in a concentration range from the lowest concentration to the *in vitro* 'cutoff'-concentration: The drug effect on each individual tumour cell line was expressed in terms of a sensitivity index SI, as defined by: $SI = AUC_x/AUC_{cut}$ (Ali-Osman *et al.*, 1983); $AUC_x$ is the area under the dose response curve of the individual tumour cell line from lowest drug concentration to the 'cut-off'-concentration and $AUC_{cut}$ is the area under the theoretical dose response curve with the identical concentration range and theoretical 100% tumour cell survival.

*Statistical analysis of sensitivity indices* Sensitivity indices of each cell line for the 3 different drugs were compared statistically; the Students' *t*-test for unrelated pairs was applied.

### Results

### *Pharmacology*

*BCNU* An *in vitro* 'cut-off'-concentration of 9.0 $\mu$mol $l^{-1}$ was found (see Table II) for a 1 h exposure.

*VM26* The *in vivo* pharmacokinetics revealed an almost linear time dependent decay of VM26 (correlation coefficient $r = -0.984$) (Figure 1). By performing a linear regression analysis, a terminal half life of 43.2 h was calculated (Table II); an *in vitro* 'cut-off'-concentration of 0.15 $\mu$mol $l^{-1}$ was found for continuous exposure (tissue). If *in vivo* plasma pharmacokinetic data would have been taken for correlation, a 'cut-off' concentration of 3.5 $\mu$mol $l^{-1}$ would have been found (plasma).

**Figure 1** *In vitro* pharmacokinetics of VM26, determined with an *in vitro* bioassay, with an initial concentration of 3.15 $\mu$mol $l^{-1}$.

*ARA-A5′P* *In vitro* pharmacokinetics followed a first order reaction: a terminal *in vitro* half life of 2.6 h could be calculated (Table II; Figure 2), which resulted in an *in vitro* 'cut-off' concentration of 75 $\mu$mol $l^{-1}$ for continuous exposure. As serum deaminase activity in individual patients (and therefore drug inactivation) may vary widely, we selected a 20% value of the calculated 'cut-off' concentration of only 15 $\mu$mol $l^{-1}$ to exclude an artificially high exposure dose.

**Table II** Synopsis of pharmacokinetic data for BCNU, VM26 and ARA-A5′P

| | *peak* $C^c$ | $T_{1/2}(h)$ | $c \times t^d$ | *'cut-off'*[c] | *Reference* |
|---|---|---|---|---|---|
| BCNU: *in vivo*: | 9.2 | 1.13 | 4.77 | — | Levin, 1975 |
| *in vitro*: | — | 0.35 | — | 9.0 (1 h) | Giannini & Levin (unpublished) |
| VM26: *in vivo*:[a] | 21.5 | 8.88 | 107.7 | — | Allen & Creaven, 1975 |
| *in vivo*:[b] | 0.39 | — | 5.25 | — | Stewart *et al.*, 1984 |
| *in vitro*: | — | 43.2 | — | 0.15 (ctd) | — |
| ARA-A5′P: *in vivo*: | 30.0 | 0.14–4.5 | 287.1 | — | LePage *et al.*, 1975 Preiksaitis *et al.*, 1981 |
| *in vitro*: | — | 2.6 | — | 75 (ctd) | — |

[a]plasma; [b]tumour tissue, (1 h/ctd): exposure 1 h or continuous; [c]$\mu$mol $l^{-1}$; [d]$\mu$mol.h$l^{-1}$.

**Figure 2** Pharmacokinetics of ARA-A5′P *in vitro*, determined by HPLC-protocol. For details see text.

*Drug effects*

Typical dose response curves obtained with BCNU, VM26 or ARA-A5′P are illustrated in Figures 3a–c. Sensitivity indices were calculated for each tumour cell line for the 3 drugs, as shown in Table I. Mean sensitivity indices of primary malignant and metastatic brain tumours may be seen in Table III, clearly metastatic brain tumours are less sensitive to all 3 agents than primary CNS neoplasms. Cell cultures with SI-values lower than 0.8, 0.5 and 0.2 are also depicted in Table IV. Sensitivity indices of each cell line for the 3 different drugs are depicted in Figure 4. If sensitivity indices of the 3 different drugs were compared on a statistical basis for each individual cell line, ARA-A5′P and VM26 were significantly more effective against brain tumour cell lines than BCNU ($P<0.001$). Differences between ARA-A5′P and VM26 were not significant. Cell cultures were also analysed for histology-related chemosensitivity (Table V): High grade malignant brain tumours generally were more resistant to the 3 drugs than low grade and infratentorial tumours.

Drug effects of ARA-A5′P on tumour colony progenitor cells were comparable to the effects seen in the micro-assay: results for 3 different cell lines may be seen in Table VI; colony formation was significantly inhibited by ARA-A5′P at a concentration of 15 μmol l$^{-1}$.

**Figure 3** Typical dose-response curves representing the effects of BCNU (a), VM26 (b) and ARA-A5′P (c) on RNA-synthesis of tumour cells.

**Table III** Synopsis of mean sensitivity indices for human brain tumour cell lines

| | $SI_{BCNU}$ | $SI_{VM26}$ | $SI_{ARA\text{-}A5'P}$ |
|---|---|---|---|
| Primary CNS tumours | 0.819 | 0.535 | 0.594 ($n=21$) |
| Metastatic tumours | 1.052 | 0.675 | 0.732 ($n=9$) |

**Table IV** Efficiency of active compounds in human brain tumour cell lines

| | | *BCNU* | *VM26* | *ARA-A5′P* |
|---|---|---|---|---|
| CNS tumours | SI < 0.8 | 7 | 18 | 15 |
| | SI < 0.5 | 4 | 7 | 7 |
| | SI < 0.2 | 3 | 5 | 4 |
| Metastatic tumours | SI < 0.8 | 2 | 6 | 6 |
| | SI < 0.5 | — | 3 | 3 |
| | SI < 0.2 | — | — | — |

**Table V** Correlation of sensitivity index with tumour histology

| | *Astrocytomas GR.IV glioblastomas* ($n=10$) | | *Low-GR. gliomas* ($n=9$) *infratentor. tumours* | |
|---|---|---|---|---|
| BCNU | SI = 0.836 | $\sigma=0.434$ | SI = 0.765 | $\sigma=0.382$ |
| VM26 | SI = 0.619 | $\sigma=0.323$ | SI = 0.421 | $\sigma=0.282$ |
| ARA-A5′P | SI = 0.68 | $\sigma=0.284$ | SI = 0.569 | $\sigma=0.345$ |

**Table VI** Comparison of ARA-A5′P activity in a colony forming assay and in the microassay

| *Cell line* | *43* | *48* | *49* |
|---|---|---|---|
| Surviving fraction (CFE) | 0.64 | 0.11 | 0.08 |
| Surviving fraction (RNA) | 0.41 | 0.16 | 0.17 |

CFE = colony forming assay; RNA = micro-proliferation assay; SF(RNA) represents RNA synthesis in remaining tumour cells relative to that of controls.

**Figure 4** Synopsis of sensitivity indices for ARA-A5'P, BCNU and VM26, with mean values (incl. s.d.) for primary CNS tumours, metastatic CNS-tumours and all cell lines.

### Discussion

This study was intended to compare the anti-brain tumour activities of BCNU, with VM26, which is currently under clinical investigation for treatment of this class of tumour, and ARA-A5'P, which has not yet been used for treatment of CNS neoplasms. An *in vitro* comparative study was performed with a microsystem that had earlier shown a good correlation between *in vitro* chemosensitivities of human malignant brain tumour cells towards BCNU and the clinical course of patients being treated with nitrosoureas (Bogdahn, 1983). In this present study 3 out of 21 primary brain tumour cell lines (14.3%) displayed high sensitivity to BCNU (SI $<$ 0.2). This proportion correponds to the number of longterm survivors among patients with anaplastic gliomas treated with BCNU (Liebermann *et al.*, 1982; Walker *et al.*, 1978, 1980). As clinical results for BCNU chemotherapy in brain tumour patients were reproduced fairly consistently in several clinical trials, and *in vitro* results were in good concordance with clinical experience, BCNU was chosen as a drug of reference in this *in vitro* investigation.

The results of previous clinical studies (Jamamoto *et al.*, 1979; Kettinger *et al.*, 1979) on the activity of VM26 on human malignant brain tumours are controversial; our results indicate high *in vitro* activity of this compound, superior to that of BCNU. Pharmacokinetic parameters for our experiments have respected the dose modifying effects of the BBB on VM26-brain tumour tissue concentrations under experimental conditions (Stewart *et al.*, 1984); the clinical mode of application (30 min infusion) should be reconsidered critically – perhaps a continuous infusion might reflect the drugs cell cycle phase specific activity in an optimal fashion.

Preliminary observations indicate anti-tumour activity of ARA-A5'P in *in vitro* systems (Bogdahn, 1983; Kufe *et al.*, 1983) and in some animal tumour systems (Bodey *et al.*, 1975, 1977). From previous studies on pharmacology (LePage *et al.*, 1975; Preiksaitis *et al.*, 1981) and on treatment of herpes simplex encephalitis (Whitley *et al.*, 1977), the drug was known to have a high permeability through the BBB ($\sim$60% of concurrent plasma levels may be found in the CSF). For these reasons ARA-A5'P seemed to be an attractive drug for further studies on activity against brain tumour cells. As variations in individual patients serum deaminase activity result in variable drug inactivation, we have employed only one fifth of the maximum achievable *in vivo* concentration time product for *in vitro* experiments; so drug activity demonstrated in our experiments will even reflect that activity that may be anticipated in those patients with extremely high serum deaminase activity and rapid drug inactivation. In this study we could clearly demonstrate that ARA-A5'P has high activity against human malignant glial tumour cells *in vitro*, since it was significantly more active ($P < 0.001$) than BCNU (see Tables I, III, IV, Figure 5). It was less active in metastatic brain tumours than in primary brain tumour cells, which was also the case for the other 2 drugs we tested. In these experiments we did not find an indication for cross-resistance of ARA-A5'P to either VM26 or BCNU. In addition, ARA-A5'P was able to inhibit growth of brain tumour colony progenitor cells in a comparable fashion to the results obtained with the microassay (Table VI).

Compared to BCNU, which has rather significant pulmonary, renal, and bone marrow toxicity (MacDonald *et al.*, 1981; West *et al.*, 1983), ARA-A5'P appears to be less toxic. Its toxic side effects (bone marrow depression, hepatotoxicity, CNS toxicity – tremor and myoclonus) were all reversible within 2 weeks at most and observed mainly under extremely high dosage (Preiksaitis *et al.*, 1981; Whitley *et al.*, 1980; Kurtz, 1975). Insufficient renal function may require dose reduction of the compound.

In conclusion, we could demonstrate high activity of ARA-A5'P in human brain tumour cell lines *in vitro*. This compound has excellent pharmacokinetic characteristics for prospective application as a chemotherapeutic agent in brain tumour therapy, it is relatively well tolerated and rather non-toxic compared to BCNU; its activity is significantly superior to BCNU in our experiments and comparable to the activity of VM26. As a result of this study we propose that ARA-A5'P should be considered for future clinical investigation in brain tumour therapy. *In vitro* results with VM26 indicate higher *in vitro* activity than found clinically so far – a proposal is made for a modified clinical drug application as a continuous application rather than a bolus infusion. We could also demonstrate that there is a proportion of metastatic brain tumours which is chemosensitive to agents active against primary CNS neoplasms; individual chemosensitivity screening might be helpful to identify these potential chemotherapy responders.

The authors acknowledge the collegial assistance of the neurosurgical (chairman: Prof K.A. Bushe) and pathology (chairman: Prof H.K. Müller-Hermelink) departments, Würzberg University, and, in addition, the neuropathological reference centre of the German brain tumour study group (Prof Kleihues, Institute of Pathology, University of Zürich, Switzerland). Special thanks is given to the excellent editorial assistance of Mrs E. Thyroff and D. Huuk. The authors are also indebted to H.T.R. Rupniak for critical review of the manuscripts.

### References

ALBERTS, B.S., CHEN, H.S.G. & SALMON, S.E. (1980). *In-vitro* drug assay: Pharmacologic considerations. In *Cloning of Human Tumour Stem Cells*, Salmon, S.E. (ed) p. 197, Allen R. Liss Inc.: New York.

ALI-OSMAN, F.S. & MAURER, R. (1983). *In-vitro* cytostatic drug testing in the human stem cell assay: A modified method for the determination of the sensitivity index. *Tum. Diag. Ther.*, **4**, 1.

ALLEN, L.M. & CREAVEN, P.J. (1975). Comparison of human pharmacokinetics of VM26 and VP16, two antineoplastic epipodophyllotoxin-glucopyranoside derivatives. *Eur. J. Cancer*, **11**, 697.

BODEY, G.P., McCREDIE, K.B., HESTER, J.P. & FREIREICH, E.J. (1977). Azacytidine and arabinosyl-adenine in the treatment of actue leukemia. *Leukemia Res.*, **1**, 257.

BODEY, G.T. GOTTLIEB, J., McCREDIE, K.B. & FREIREICH, E. (1975). Adenine-arabinoside in cancer chemotherapy. In *Adenine-arabinoside: An Antirival Agent*, Parvane-Langstone, D. *et al.* (eds) p. 181. Raven Press: New York.

BOGDAHN, U. (1983). Chemosensitivity of human malignant brain tumours. *J. Neuro-oncol.*, **1**, 149.

BOGDAHN, U., RUPNIAK, H.T.R., ALI-OSMAN, F. & ROSENBLUM, M.L. (1985). Characterization of human malignant brain tumour cells *in vitro* and comparison of three different *in vitro* assays to determine their sensitivity to BCNU. In *Chemotherapy of Gliomas*, Voth, D. (ed) p. 321. Walter de Gruyter, Berlin, New York.

DeTRIBOLET, N., CARREL, S. & MACH, J.P. (1984). Brain tumor associated antigens. *Progr. Exp. Tumor Res.*, **27**, 118.

ENG, L.F., VANDERHAEGEN, J.J., BIGNAMI, A. & GERSTL, B. (1971). An acidic protein isolated from fibrous astrocytes. *Brain Res.*, **28**, 351.

JAMAMOTO, H., SHITARA, N., TAKAKURA, K. & SANO, K. (1979). Recruitment chemo-radiotherapy with VM26 (epipodophyllotoxin) for induction treatment of malignant brain tumors. *Acta Neurochir.* Suppl., **28**, 616.

KETTINGER, A., LEMON, H.M. & FOLEY, J.F. (1979). VM26 as a second drug in the treatment of brain gliomas. *Cancer Treatment Rep.*, **63**, 511.

KUFE, D.W., MAJOR, P.B., MUNROE, D., EGAN, M. & HERRICK, D. (1983). Relationship between incorporation of 9$\beta$-D-arabinofuranosyl-adenine in L1210 DNA and cytotoxicity. *Cancer Res.*, **43**, 2000.

KURTZ, S.M. (1975). Toxicology of adenine-arabinoside. In *Adenine-arabinoside – An Antirival Agent*, Parvan-Longstone, D. *et al.* (eds) p. 145. Raven Press: New York.

LePAGE, G.A., NAIK, S.R., KATAKKAR, S.B. & KHALIQ, A. (1975). $\beta$-D-arabinofuranosyl-adenine-5-phosphate metabolism and excretion in humans. *Cancer Res.*, **35**, 3036.

LEVIN, V.A., HOFFMAN, W. & WEINKAM, R.J. (1978). Pharmacokinetics of BCNU in man: A preliminary study of 20 patients. *Cancer Treatment Rep.*, **62**, 1305.

LIEBERMANN, A.N., SUN HO PHO, RANSOHOFF, J., WIESE, A., GEORGE, A., GORDON, W. & WALKER, R. (1982). Longterm survivors among patients with malignant brain tumors. *Neurosurgery*, **10**, 450.

MacDONALD, J.S., WEISS, R.W., POSTER, D. & DUQUE-HAMERSHAMB, L. (1981). Subacute and chronic toxicities associated with nitrosourea therapy. In *Nitrosoureas – Current Status and New Developments*, Prestayko, A. (ed) p. 145. Academic Press: New York.

MÜLLER, W.E.G., ZAHN, R.K., BITTLINGMAIER, K. & FALKE, D. (1977). Inhibition of herpes virus DNA-synthesis by 9$\beta$-D-arabinofuranosyl-adenine in cellular and cell-free systems. *Ann N.Y. Acad. Sci.*, **184**, 34.

PREIKSAITIS, J.K., LANK, B., NG, D.K., BROX, L., LePAGE, G.A. & TYWELL, D.J.L. (1981). Effect of liver disease on pharmacokinetics and toxicity of 9-$\beta$-D-arabinofuranosyl-adenine-5-phosphate. *J. Infect. Dis.*, **144**, 358.

ROSENBLUM, M.L., GEROSA, M.A., WILSON, C.B., BARGER, G.R., PERTUISSET, G.S., DeTRIBOLET, N. & DAUGHERTY, D. (1983). Stem cell studies of human malignant brain tumours. *J. Neurosurg.*, **58**, 170.

SEEGER, R.C., DANON, Y.L. & RAINER, S.A. (1982). Definition of a Thy-1 determinant on human neuroblastoma, glioma, sarcoma, and teratoma cells with a monoclonal antibody. *J. Neurol.*, **128**, 983.

STEWART, D.J., RICHARD, M.T., HOGENHOLZ, H., DEMMERY, J., NONDY, B., PRIOR, J. & HOPKINS, H.S. (1984). Penetration of Teniposide (VM26) into human intracerebral tumours. *J. Neuro-oncol.*, **2**, 315.

THOMAS, D.G.T., DARLING, J.L., FRESHNEY, R.I. & MORGAN, B. (1979). *In-vitro* chemosensitivity assay of human glioma by scintillation autofluorography. In *Multidisciplinary Aspects of Brain Tumour Therapy*, Paoletti, G. (ed) p. 19. Elsevier-North Holland, Amsterdam.

TOFILON, P.J., WILLIAMS, M.E. & DEEN, D.F. (1983). Nitrosourea induced sister-chromatid exchanges and correlation to cell survival in 9L-rat brain tumor cells. *Cancer Res.*, **43**, 473.

WALKER, M.D., ALEXANDER, E.J., HUNT, W.E., McCARTY, C.S., MAHALEY, M.S., GEHAN, E.A. & STRIKE, T.A. (1978). Evaluation of BCNU and/or radiotherapy in treatment of anaplastic gliomas. *J. Neurosurg.*, **49**, 333.

WALKER, M.D., GREEN, S.B., BYAR, D.P. *et al.* (1980). Randomized comparison of radiotherapy and nitrosouresas for the treatment of malignant glioma after surgery. *New Engl. J. Med.*, **303**, 1323.

WEST, C.R., AVELLANOSA, A.M., BARNA, N.R., PATEL, A. & HONG, C.I. (1983). Intraarterial 1,3-Bis(2-Chlorethyl)-1-Nitrosourea (BCNU) and systemic chemotherapy for malignant gliomas: A follow-up study. *J. Neurosurg.*, **13**, 420.

WHITLEY, R.J., SOONG, S.J., DOLIN, R., GALASSO, G.J., COHEN, L.T. & ELFORD, C.H. (1977). The collaborative study group: Adenin-arabinoside therapy of biopsy-proved herpes simplex encephalitis. *New Engl. J. Med.*, **297**, 289.

WHITLEY, R.J., TUCKER, B.C., KINKEL, A.W. & 6 others. (1980). Pharmacology, tolerance and antiviral activity of Vidarabine-monophosphate in humans. *Antimicrob. Chemother.*, **18**, 409.